# Reduction of the onset response in kilohertz frequency alternating current nerve block with amplitude ramps from non-zero amplitudes

**DOI:** 10.1186/s12984-019-0554-4

**Published:** 2019-06-28

**Authors:** T. L. Vrabec, T. E. Eggers, E. L. Foldes, D. M. Ackermann, K. L. Kilgore, N. Bhadra

**Affiliations:** 1 MetroHealth Medical Center, Cleveland, OH USA; 2 Dept of Biomedical Engineering, Case Western Reserve University, Cleveland, OH USA; 3 College of Health Solutions, Arizona State University, Tempe, AZ USA; 4 CEO, Presidio Medical, Inc, San Francisco, USA; 5 Louis Stokes Cleveland Department, Veterans Affairs Medical Center, Cleveland, OH USA

**Keywords:** Electrical nerve block, Functional electrical stimulation, Stroke rehabilitation, Spasticity

## Abstract

**Background:**

Kilohertz frequency alternating current (KHFAC) waveforms reversibly block conduction in mammalian peripheral nerves. The initiation of the KHFAC produces nerve activation, called the onset response, before complete block occurs. An amplitude ramp, starting from zero amplitude, is ineffective in eliminating this onset activity. We postulated that initiating the ramp from a non-zero amplitude would produce a different effect on the onset.

**Methods:**

Experiments were conducted in an in vivo rat model. KHFAC was applied at supra block threshold amplitudes and then reduced to a lower sub block amplitude (25, 50, 75 and 90% of the block threshold amplitude). The amplitude was then increased again to the original supra block threshold amplitude with an amplitude ramp. This ramp time was varied for each of the amplitude levels tested.

**Results:**

The amplitude ramp was successful in eliminating a second onset. This was always possible for the ramps up from 75 and 90% block threshold amplitude, usually from 50% but never from 25% of the block threshold amplitude.

**Conclusions:**

This maneuver can potentially be used to initiate complete nerve block, transition to partial block and then resume complete block without producing further onset responses.

## Background

Kilohertz frequency alternating currents (KHFAC) when applied to peripheral nerves with an encircling cuff electrode produces a rapid and reversible conduction block with minimum side effects [1–6]. KHFAC nerve block offers an attractive alternative treatment modality for blocking unwanted nerve activity. KHFAC is currently being explored in a variety of preclinical work, including treating facial palsy [7] and blocking autonomic activity [8], and is employed in clinical trials for spinal cord stimulation [9], vagal block for obesity control [10] and for neuroma pain [11]. One characteristic of KHFAC block is the short burst of neuronal firing when the block is first initiated, which has been demonstrated experimentally in mammalian motor nerves [12] as well as simulation studies [13]. This activity, termed the ‘onset response’ [2, 4, 14], is undesirable for clinical applications. Onset activity is comprised of two phases, termed Phase I (which lasts < 1 s) and Phase II (can last up to 30s). Our laboratory has been studying various methods to eliminate or reduce this onset activity, and has found techniques to reduce Phase II activity [15]. However, the presence of the Phase I onset is still an impediment to generalized clinical applications.

Some methods have been developed to mitigate the onset response [16–18]. One suggestion was that a slowly increasing amplitude ramp would eliminate the onset. We have previously shown that an amplitude ramp starting from zero amplitude not only fails to eliminate the onset response but in fact makes it more intense [19]. This onset is likely due to the amplitude crossing the activation threshold, or the minimum current/voltage needed to activate the nerve. Interestingly, activation of the nerve typically occurs around 30 to 40% of KHFAC block threshold (BT) [14], which is defined as the minimum voltage/current needed to completely block the nerve. We therefore postulated that an amplitude ramp that did not start from zero amplitude but from above this activation threshold might show a different behavior. In the amplitude range above activation but below block, the nerve can be in a state of partial block, which means that some but not all of the nerve fibers may be blocked while others conduct normally.

We envisioned a scenario where the block is turned on at or above block threshold, with an initial onset response (which could be mitigated by other techniques), and then the block is transitioned between states of complete block and partial (or no block), depending on the requirements of the application. *We postulated that an amplitude ramp that started from an amplitude above the activation threshold might allow this transition to occur without producing a second onset response.* In this way, the KHFAC would be kept continuously on at either complete block or partial to no block levels yet could be switched between those two levels without eliciting any new onset responses. Since the KHFAC would not be completely turned off, the onset responses that would typically occur every time the KHFAC was cycled from completely off to full block would be avoided. This technique could be used in the rehabilitation of stroke patients to control muscle spasticity. This paper describes our experimental investigation of this postulate. Preliminary data on this study has previously been published [20].

## Methods

Acute experiments were performed in adult Sprague-Dawley rats. All protocols involving animal use were approved by our institutional animal care and use committee. Six animals were tested, with the first animal providing preliminary data for optimization of the protocol and the other five providing randomized data for analysis. The animals were anesthetized with intraperitoneal injections of Nembutal (Pentobarbital sodium). The left hind leg was shaved and an incision was made along the posterior aspect of the leg and thigh. The sciatic nerve was exposed. The common peroneal and sural nerves were severed. The gastrocnemius-soleus muscle complex was dissected, and the calcaneal (Achilles) tendon was severed from its distal attachment. The ipsilateral tibia was stabilized to the experimental rig via a clamp, and the calcaneal tendon was tethered to a force transducer (Entran, resolution 0.005 Newtons) with 1–2 N of passive tension [4]. Animals were euthanized after the experiment with Fatal Plus.

**Fig. 1:**
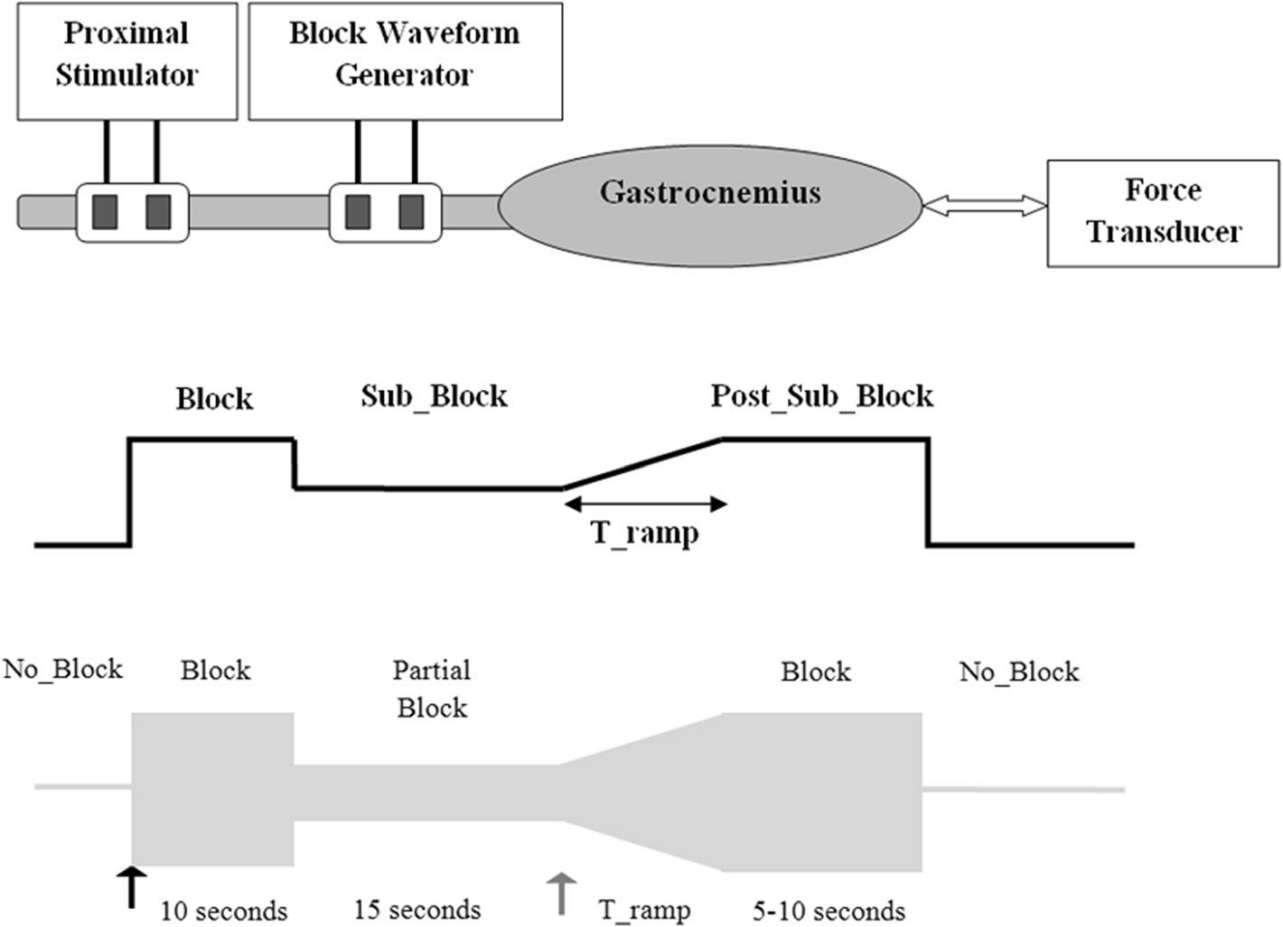
Experimental setup showing the block electrode on the sciatic nerve and proximal nerve stimulation. Force is recorded from the muscle. The waveform is shown below. The upper trace shows the control signal from Labview® with the three amplitude zones and the ramp. The lower trace shows the KHFAC output. The black arrow points to onset when the KHFAC is first turned on. The grey arrow points to where onset occurs for a step change transition from Sub_Block to Post_Sub_Block. This onset can be eliminated by the use of a ramp (T_ramp)

Two nerve cuff electrodes were placed on the sciatic nerve (Fig. 1). Both were bipolar J-shaped silastic nerve cuff electrodes [4, 21] with 3 mm × 1 mm rectangular platinum contacts [4, 21]. The proximal electrode was used to generate gastrocnemius muscle twitches with the delivery of 1 Hz, 20 μs, supramaximal (typically 300–500 μA) cathodic pulses. These pulses were delivered using a Grass S88 stimulator (Grass Technologies) with an isolated current-controlled output stage (Grass Technologies Optical Isolator). The distal electrode was used to deliver the blocking waveform from a voltage controlled waveform generator (Model 395, Wavetek) with 3 μf capacitors in series to eliminate any direct current (DC) offsets. All KHFAC ramp experiments were performed using a 20 kHz sinusoidal blocking waveform. Labview® software controlled the waveform generator and modulated the output to obtain specific ramps as desired. The data sampling rate for the force transducer was 1000 Hz.

At the beginning of each experiment, supramaximal stimulation through the proximal electrode was verified by increasing the pulse amplitude until the peak twitch force plateaued. Stimulation through the proximal electrode was used throughout the experiment to determine if the block was effective. When block was 100% effective, supramaximal proximal stimulation did not produce any twitch responses.

Block threshold, defined as the lowest KHFAC amplitude that produces complete (100%) conduction block [4], was determined in each experiment. Block threshold was determined by first delivering KHFAC at 20 kHz at 10 V_pp_ during proximal stimulation at 1 Hz. This amplitude and frequency of KHFAC was always sufficient to produce a 100% block. Once the onset response subsided, complete block was verified by the absence of muscle twitches. The KHFAC voltage was then lowered in a stepwise manner in one volt decrements until twitches started to appear. The lowest voltage that yielded a complete motor block was designated as the block threshold [4, 22].

The response of the nerve-muscle system to amplitude transitions was evaluated using the waveform depicted in Fig. 1 (lower figure). The three phases in each trial have been termed; “Block”, “Sub_Block” and, “Post_Sub_Block” (Fig. 1). Each trial began with proximal stimulation at 1 Hz and maximal twitch responses were observed. After four to five twitches, KHFAC at 20 kHz and an amplitude that was 125% of block threshold was delivered through the block electrode. The KHFAC produced an initial onset response which subsided within 10 s, revealing a loss of proximally-generated muscle twitches, thus verifying complete block. After 10 s of KHFAC at 125% of block threshold, the block amplitude was reduced to one of four Sub_Block threshold levels (25, 50, 75% or 90% of block threshold), where it was maintained for 10 s. Note that reduction of the KHFAC amplitude does not cause any secondary onset responses. Once the KHFAC was reduced below block threshold, proximally-generated muscle twitches could again conduct through the block region. The peak level of the muscle twitches during this period was recorded and compared to the proximal muscle twitches obtained prior to the initiation of KHFAC block. This provided a direct measure of the percentage of block at each of the Sub_Block threshold amplitudes. *It should be noted that the percentage of block is not equivalent to the Sub_Block threshold amplitude.* Thus, 75% of block threshold may result in 50% block and 50% of block threshold may result in 0% block. After the percentage of block during the Sub_Block phase was recorded, the proximal stimulation was turned off to allow unobstructed recording of any further onset responses. After 15 s of subthreshold block, the KHFAC amplitude was transitioned back to 125% of block threshold over a time period of T_ramp, where it was maintained for another 5 to 10 s (Post_Sub_Block phase) before the KHFAC was turned off and the trial terminated.

**Fig. 2:**
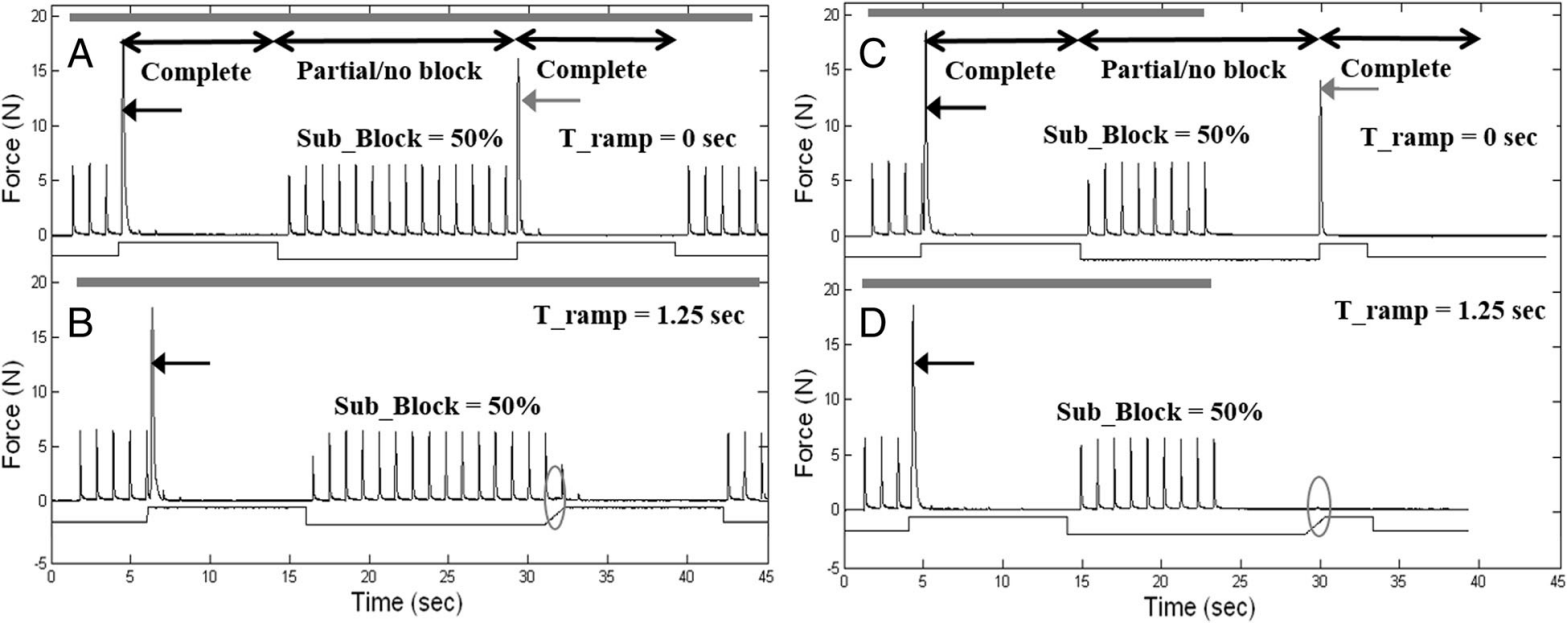
Data from the preliminary animal showing the proximal stimulation maintained throughout the trials (**a**, **b**, gray bars at top of each figure) and matching trials where the proximal stimulation was stopped before the ramp (**c**, **d**). At a Sub_Block level of 50% there is no block (**a**, **b**, **c**, **d**). Zero ramps produce secondary onset (**a**, **c**, grey arrows) and are the control trials in every group of trials. A ramp of 1.25 s still produces a tiny secondary onset that is partially obscured by the proximal twitches (**b**) but is visible and measurable in “D” (circles). This figure shows why proximal stimulation was stopped before the ramp for the definitive trials. It shows that complete block was obtained in the Post_Sub_Block phase and demonstrates that the proximal stimulation does not enhance the onset response (**b**, **d**), but in fact can obscure it. The decreasing twitch heights seen in B are examples of partial block, in which only some of the motor fibers are blocked

In preliminary experiments, the proximal stimulation was not turned off during the Post_Sub_Block phase (as shown in Fig. 2: a, b). These trials demonstrated that complete block was again established in the Post_Sub_Block phase, when the KHFAC was turned on again at 125% of block threshold. They also demonstrate that the proximally stimulated twitches did not enhance the second onset response. However, the presence of these twitches made it difficult to accurately measure small onset responses during the ramp due to overlap of twitches and onset responses, leading to inaccurate estimations of the onset (Fig. 2 b, circle). Therefore, in the definitive experiments, the proximal stimulation was stopped before the ramp was started (Fig. 2: c, d).

A goal-directed search was performed to establish the T_ramp value that did not produce a second onset response using multiple trials. The goal-directed search trials began with a step transition (T_ramp = 0 s) to allow quantification of the onset response without a ramp. This acted as a control trial for every cluster of trials for each specific Sub_Block threshold amplitude and showed an onset response to the step. This was followed by a trial with a T_ramp of 2.5 s. If an onset occurred, T_ramp was increased and retested until no onset occurred. The T_ramp values tested were 5, 10, 15, 20, 25 and, 30 s, always in that order. Once a T_ramp value was found that did not produce an onset response during the transition, the minimum T_ramp was determined using a binary search pattern at a ramp time resolution of 0.3125 s. The maximum T_ramp tested was 30 s (10 s for the first animal). If the maximum T_ramp was reached and a second onset response was still present, it was considered a failure of the ramp maneuver for that particular Sub_Block amplitude. The order in which the four levels of sub-block threshold were tested was randomized, and the set of four randomized levels was repeated three times in each definitive animal.

## Results

**Fig. 3:**
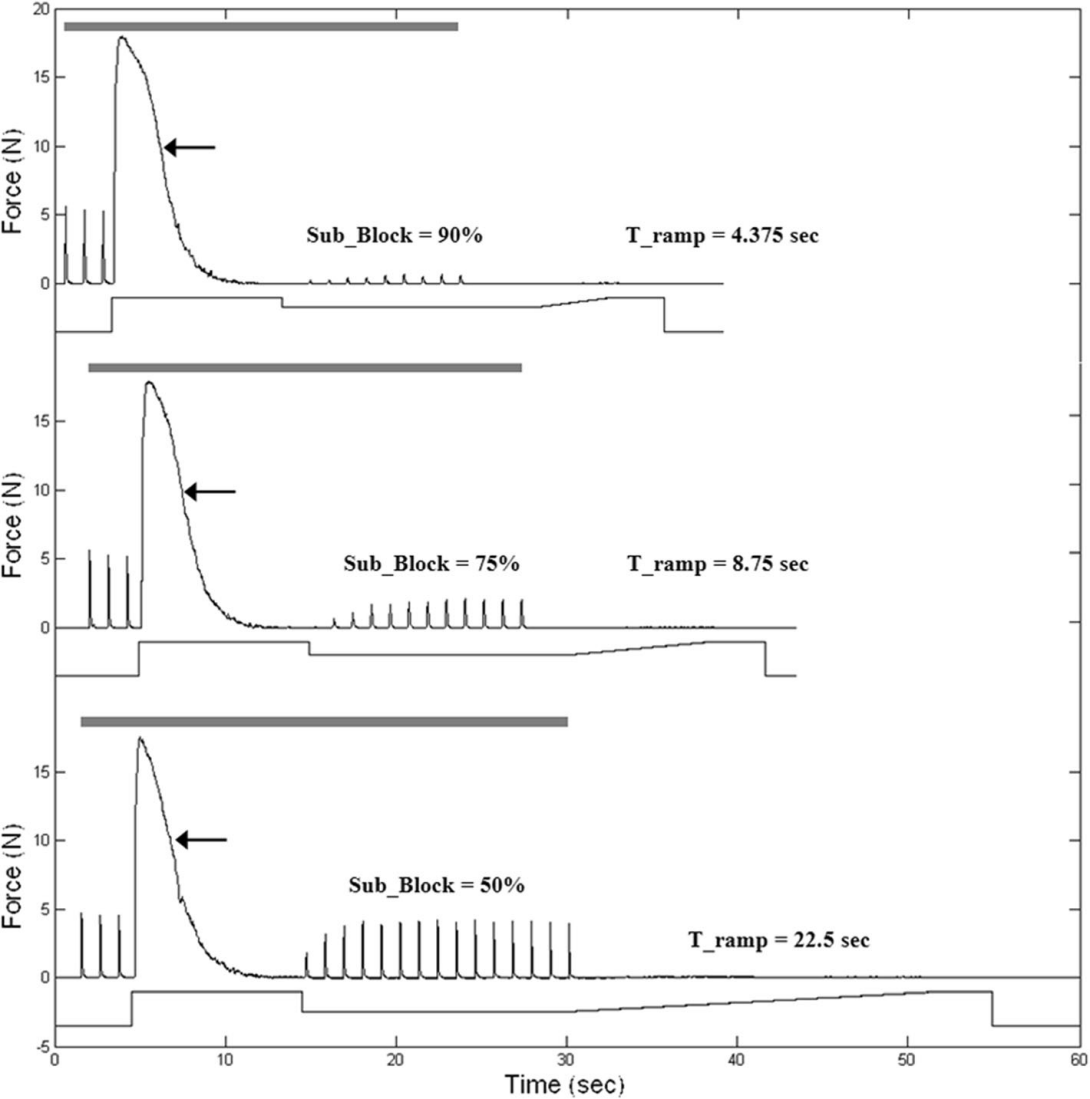
Comparison of minimum ramp times required for elimination of second onset: Each top trace is the force, and the bottom trace is the amplitude control waveform. Black arrows show onset at the start of KHFAC. The gray bar shows proximal stimulation which was run until ~ 23–25 s. Different Sub_Block values were evaluated and the smallest ramp time to produce no onset is displayed in this graph. Higher Sub_Block values require shorter ramp times to eliminate secondary onset

Complete block was obtained in all animals at 20 kHz. The range of block thresholds was 4.6 to 7.1 V_pp_. A voltage controlled source with blocking capacitors was used to minimize the risk of damage due to unintended DC [23]. Three repeats were completed in all animals except animal 2 where only two repeats could be completed. The initial instantaneous start of the KHFAC at “Block” caused an onset in every trial (short black arrows in all figures). There were no onset responses during the transition from “Block” to the subthreshold amplitude of Sub_Block. During the Sub_Block phase, varying degrees of partial block occurred. The partial block level depended on the subthreshold amplitude, with lower amplitudes resulting in less block (Fig. 3). As the amplitude was ramped from the Sub_Block to the Post_Sub_Block value, there was always an onset if T_ramp was zero, i.e. a step function (grey arrows, Fig. 2). When the blocking waveform was turned off, block ended and the proximal stimulation (if turned on) could once again conduct through the block region, causing twitches to occur (Fig. 2: a, b).

While a step increase in the KHFAC amplitude at the transition from Sub_Block to Post_Sub_Block caused an onset, this onset could be eliminated *under certain circumstances* by using a ramp. A successful outcome depended on the subthreshold amplitude that was chosen during the Sub_Block phase and the ramp time. Subthreshold values closer to block threshold required shorter ramps. Figure 3 shows the minimum ramp time needed to eliminate onset for Sub_Block amplitude values of 90, 75 and 50%. The amount of ramp time needed to eliminate onset for 50% as compared to 90% is more than 5 times as long in this example. For Sub_Block amplitude values of 25%, the onset *could not* be eliminated with a ramp in any trial (the maximum ramp time used in this study was 30 s).

It is also notable that the onset responses are more prolonged as the ramp times are increased for the 25% amplitude value. Table 1 summarizes the number of successful searches/total searches for a successful minimum ramp time for each animal for each Sub_Block amplitude value. All minimum ramp searches for 90 and 75% were successful in achieving a no onset response during the transition back to block, whereas 71% of the runs for the 50% sub-block amplitude were successful, and 0% of the runs at 25% sub-block amplitude were successful. Table 1 also shows the block thresholds for each of the rats.

**Table 1:** Ratio of successful searches/total searches and block thresholds for all definitive animals

Animal #	Sub_Block Percentage				BT (Vpp)
	25%	50%	75%	90%	
1	0/3	3/3	1/1	3/3	4.6
2	0/2	1/2	2/2	2/2	7.1
3	0/3	2/3	3/3	3/3	6.8
4	0/3	3/3	3/3	3/3	5.0
5	0/3	1/3	3/3	3/3	6.0

**Fig. 4:**
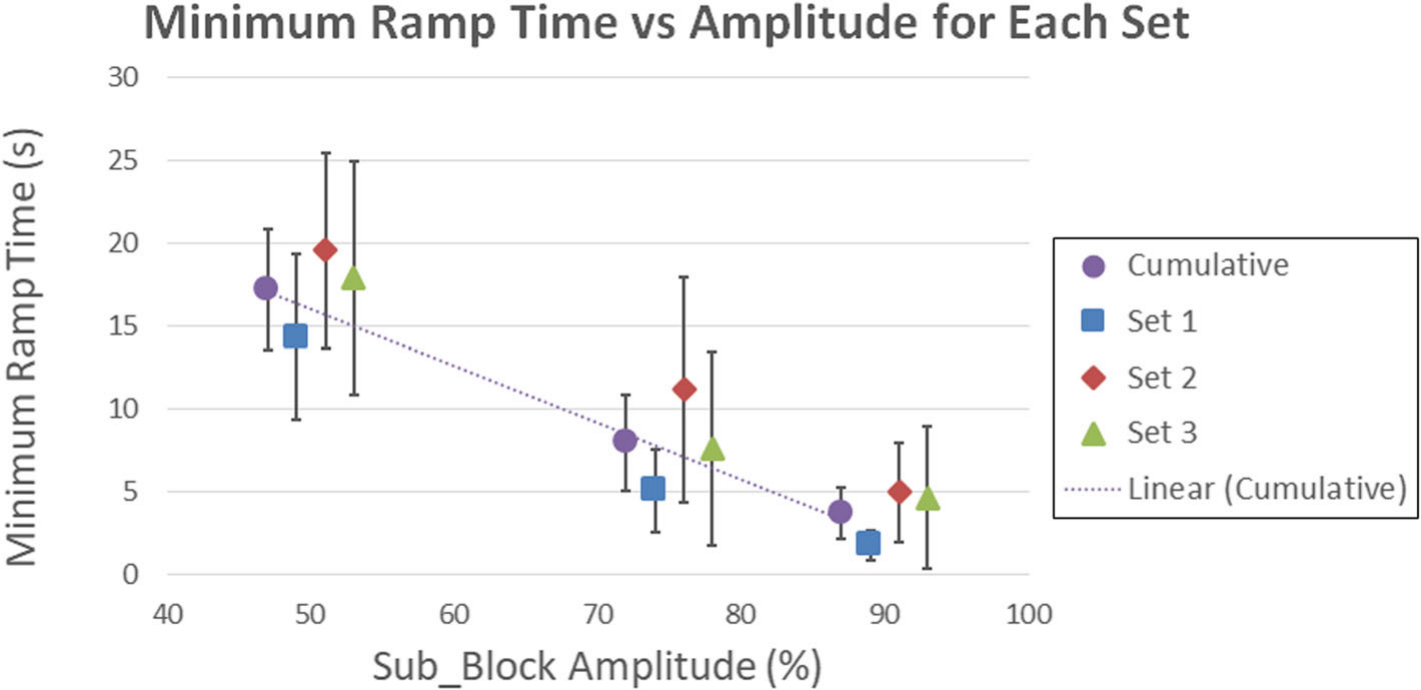
Minimum successful ramp times at each subthreshold value for each set and the cumulative values for each set (mean ± SEM). Also shown is the best fit line for the cumulative data. The data shows an increasing ramp time for lower Sub_Block amplitudes. The ramp times also increased between set 1 and set 2 in all amplitudes and animals

There is variability between animals resulting in a range of minimum T_ramp values for the different Sub_Block values, which are shown in Fig. 4. However, successful no-onset block was still obtained within our maximum T_ramp value of 30 s for Sub_Block values of 50, 75 and 90%. The cumulative fit line in Fig. 4 showed a statistically significant slope (*p* < 0.01, t-test), demonstrating the linear relationship between minimum ramp times and subthreshold amplitude.

It would be desirable in many cases to be able to transition from 0% block to 100% block without producing an onset. We evaluated all trials to determine which trials produced 0% block during the Sub_Block phase. Only one trial with a Sub_Block of 75% produced 0% block and could be transitioned back to block without a second onset. Twenty trials with a Sub_Block of 50% were similarly successful (Fig. 2f), with such successful trials occurring in 4 out of 5 rats. Table 2 shows the mean and standard deviation (SD) of the percentage of block versus the Sub_Block amplitude percentage used. Data from trials with 25% Sub_Block amplitude are not shown as they all resulted in 0% block.

**Table 2:** Percentage of block (mean ± SD) for each subthreshold block amplitude for each animal

Animal #	Sub_Block Percentage		
	50%	75%	90%
1	4.1 ± 1.4	18.1 ± 7.6	91.2 ± 1.6
2	12.3 ± 4.6	93.4 ± 1.2	100 ± 0
3	12.7 ± 4.2	57.7 ± 5.0	81.6 ± 3.0
4	2.4 ± 1.3	41.8 ± 18.0	82.3 ± 9.5
5	1.0 ± 2.1	17.4 ± 6.6	78.3 ± 6.4

## Discussion

These experimental results validate our postulate of using subthreshold amplitude KHFAC during periods where block is not required and then ramping the amplitude up to complete block amplitude. In all animals, complete block could be obtained at 20 kHz at block thresholds that were similar to previous studies [4, 17]. The initial period of complete block started with an onset response [4]. The complete block could then be transitioned to partial block or no block by reducing the KHFAC amplitude. This reduction did not produce an onset response since in KHFAC the onset response is only produced during positive gradients in injected charge to the nerve. Following this period of partial or no block, the KHFAC amplitude could be successfully increased to produce complete block again. At this transition, the second onset response that would be produced could be eliminated by using an amplitude ramp.

Our results show that the nerve response is substantially different when the KHFAC is transitioned from block to subthreshold and back to block again when compared to the transition from zero amplitude to block threshold [19]. In contrast to ramps starting at zero amplitude, longer ramp times were associated with a smaller secondary onset response in all cases. This time dependence could relate to a maximum slope, or equivalently a maximum increase in injected charge per phase, that can occur without activating the nerve. When the amplitude is ramped from zero amplitude to block amplitude, the amplitude has to cross the nerve activation threshold level which produces action potentials (the onset response). The onset response is intensified using ramps, likely due to the axons taking longer to cross this activation threshold. The results in this paper show that when the amplitude of the KHFAC is 50 to 90% of block threshold, a linear ramp transition can be accomplished without producing an onset. We hypothesize that this effect is because the amplitude ramp no longer crosses the activation threshold level and instead traverses a “non-firing steady state” region [14]. This region has some unusual properties because the nerve membrane experiences rather significant swings in potentials without activating or blocking neural activity. Further study is warranted into this unique state of nerve inactivity.

The data presented in this manuscript showed considerable variation across animals for the minimum ramp times and percentage of block. As noted in Fig. 4, the minimum ramp times increased from set 1 to set 2 in all animals. This increase is likely due to the increased time under anesthesia, either directly due to the effect of the anesthesia on nerve conduction or due to the general decline in nerve viability over the course of the experiment. However, ANOVA revealed that these set differences were not significant (*p* > 0.05). Another factor contributing to the variance in ramp times is the block threshold itself; higher block thresholds required longer ramp times than lower block thresholds. This relationship suggests that the slope of the ramp is important in determining the necessary ramp time. There is also large variability in the percentage of block for a given Sub_Block percentage, particularly for the 75% seen in Table 2. We hypothesize that this variability is due to the nature of partial block. Block percent follows a sigmoid function [4], similar to the nerve activation function. The 75% Sub_Block amplitude likely falls on the steep portion of this function, increasing the variability seen in the block percent.

We expect that there is a maximum duration over which the subthreshold KHFAC can be maintained without affecting nerve conduction. Based on our work with prolonged KHFAC [24], it is possible that a reversible block carry over effect could occur after a period of hours. However, that study was conducted with KHFAC at or above block threshold amplitudes. We postulate that Sub_Block threshold amplitudes could lead to even longer durations of KHFAC applications before a carry over effect is observed. In a future study, we expect to evaluate the parameters that affect prolonged subthreshold KHFAC delivery.

The method presented can be reliably used to transition from a partially blocked state to a fully blocked state without producing an onset response. However, our results show that it was not possible to transition from a Sub_Block value that consistently produced 0% block and then transition back to 100% block in every animal. In our series, this could be achieved in 4 of 5 animals. It may be that the use of Sub_Block values between 50 to 75% would improve the likelihood of achieving “no block to full block” transition, and further testing in this range should be performed in the future.

This work is limited by the fact that all data is acquired in acute experiments from anaesthetized animals. It is possible that the pentobarbital sodium affected the nerve conduction characteristics (noted previously), thereby altering the onset response and influencing the ramp times. Lastly, this work only evaluated the onset response in motor fibers; it is unclear if this effect holds for sensory or autonomic fibers as well.

## Conclusions

It has been demonstrated that subthreshold ramping is a feasible solution for producing no onset during prolonged use of KHFAC block, following the initial onset. Amplitudes above 50% block threshold can reliably be ramped to 125% without causing onset while amplitudes below 25% will always produce an onset. As the subthreshold percentage is increased, the minimum ramp time to produce onset is decreased. Subthreshold amplitudes of 50% of block threshold can result in almost complete absence of block. This implies that block could be gated on or off even though the KHFAC is constantly on, and may provide a means of maintaining no-onset KHFAC block.

## Data Availability

Data from this study will be made available upon reasonable request.

## Abbreviations

BT: Block threshold
DC: Direct current
KHFAC: Kilohertz frequency alternating current
SD: Standard deviation
